# Conformational plasticity in GDAP1’s GST-like domain enables thapsic acid recognition

**DOI:** 10.1016/j.jbc.2026.113378

**Published:** 2026-07-28

**Authors:** Nicholas T. Arredondo, Michael A. DaSilva, Maya R. Brown, Marios Gavrielatos, Sarah M. Helsel, Matthew R. Googins, Zoe Freyberg, David R. Koes, Kirill I. Kiselyov, Andrew P. VanDemark

**Affiliations:** 1 Department of Biological Sciences, University of Pittsburgh, Pittsburgh, Pennsylvania, USA; 2 Department of Computational and Systems Biology, University of Pittsburgh, Pittsburgh, Pennsylvania, USA

**Keywords:** GDAP1, glutathione, Charcot-Marie-Tooth (CMT), mitochondria, oxidative stress

## Abstract

The GST superfamily of proteins canonically use a large central active site to recognize and detoxify the cell from a range of damaged lipids and xenobiotics. The mitochondrial GST-like protein, GDAP1, was proposed to bind the dicarboxylic fatty acid, thapsic acid, through a non-canonical binding pocket, similar to a GST L-site, allowing GDAP1 to adopt an unusually open conformation, and leaving the function of the canonical active site and an adjacent feature called the alpha-loop unclear. Here, we report the biochemical impact of mutants in all of GDAP1’s canonical domains (G-site, H-site, alpha-loop, and putative L-site), revealing that thapsic acid binding activity is actually housed within the canonical active site pocket. Our biochemistry, combined with structural predictions and modeling strongly suggests that movements in the alpha-loop and G-site effectively remodel the canonical GDAP1-binding pocket to facilitate interactions with thapsic acid. Further, mutants in the canonical active site eliminated the cytoprotective effects of wild-type GDAP1. Together, these findings establish GDAP1 as a conformationally dynamic GST-like protein in which coordinated alpha-loop and G-site movements remodel the canonical active site to enable lipid recognition, coupling its biochemical activity to mitochondrial membrane integrity and redox homeostasis. Furthermore, we provide an extensive map of residues that coordinate lipid binding by GDAP1.

Charcot-Marie-Tooth (CMT) disease is the most common inherited peripheral neuropathy. This genetically heterogeneous disease results from mutations within an ensemble of genes (1, 2, 3), many of which affect mitochondrial function. *GDAP1* (ganglioside-induced differentiation-associated protein 1) is an outer mitochondrial membrane protein highly expressed in peripheral neurons (4, 5, 6) and mutated in CMT subtypes 2K and 4A (6, 7). Studying *GDAP1* presents a unique opportunity to learn about CMT pathogenesis, as more than 70 single missense mutations in *GDAP1* have been identified in CMT patients (8, 9, 10); however, direct connections between features of the GDAP1 protein and its biochemical and cellular functions have been unclear.

Previous reports associate GDAP1 with a variety of mitochondrial and redox-sensitive functions, including regulation of oxidative stress, mitochondrial membrane potential, mitochondrial fission, and ion transport (4, 11, 12, 13). Overexpression of WT but not a CMT mutant GDAP1, increased mitochondrial membrane potential and decreased oxidative stress indicating improved mitochondrial fitness (12). In earlier studies, GDAP1 overexpression in human cells resulted in hyperfragmented mitochondria, whereas GDAP1 knockdown caused hyperfused mitochondria (4), consistent with a role in regulating mitochondrial network dynamics. Our own data show mitochondrial swelling and perinuclear clustering under overexpression conditions, while moderate expression fine-tunes the response of the mitochondrial shape and network to oxidative stress (14). Sequences within the GDAP1 protein responsible for these functions and the mechanisms by which they are driven are unknown.

Both primary sequence and structural homology have identified GDAP1 as a novel member of the glutathione S-transferase (GST) superfamily of detoxifying enzymes (14, 15, 16, 17, 18, 19). Canonical GSTs have a composite active site formed in part by a highly conserved glutathione (GSH)-binding “G-site” which adopts a thioredoxin fold also seen in deiodinases (20) and peroxidase (21) enzymes; and a less conserved, helical “H-site” which recognizes hydrophobic electrophilic substrates (22). GSTs recognize a broad range of substrates, such as oxidized lipids (23), hormones (24), fatty acids (25), and numerous xenobiotics (26, 27). Canonical GSTs utilize G- and H-sites to bind and position both co-substrates near a catalytic tyrosine or serine, facilitating GSH conjugation to these substrates (28) and subsequent export out of the cell *via* MRP transporters (29). The preponderance of data, however, suggests that GDAP1 does not harbor GSH binding or GST conjugation activities (14, 15, 19), leaving the biochemical function of GDAP1’s active site unclear.

In addition, many GSTs have evolved a variety of non-enzymatic Ligandin-sites or “L-sites” which are distinct from the canonical GST active site and function in ligand transport and storage (30, 31). Some L-sites overlap with the H-site (32), while others are found at the dimer interface between GST monomers (31, 33, 34). L-sites that overlap with the H-site provide non-competitive inhibition of the GST active site (35). GDAP1’s proposed L-site is formed by both G- and H-sites but on the opposite face as its canonical GST-like active site (15). Previously, a metabolite screen identified hexadecanedioic acid (thapsic acid, TA), a 16-carbon dicarboxylic saturated fatty acid, as a ligand for GDAP1 that interacts at the proposed L-site (15). TA has been shown to increase mitochondrial respiration and mitochondrial calcium release in rat liver mitochondria, but the mechanism and direct connection between GDAP1 and TA has not been established for these functions (36, 37).

Beyond the GST-like core, the GDAP1 protein also contains an insertion between helices 4 and 5, called the alpha-loop, formed by residues 153 to 200 (14, 18). The alpha-loop has long been recognized as a unique signature motif for GDAP1 (14, 18, 38), and structural studies of a longer GDAP1 fragment containing the alpha-loop suggested that it forms an extended helical domain pointing away from the rest of the protein (15) and that this conformation did not change in the presence of TA (15). Paradoxically, a decrease in GDAP1’s hydrodynamic radius was reported upon TA binding, and our previous data demonstrated that the alpha-loop is required for binding of the pan-GST inhibitor ethacrynic acid to mouse GDAP1 (14). Molecular dynamics simulations and our initial structural studies both suggest alpha-loop movements relative to the GST-like core (14, 17). These results lead us to hypothesize that the alpha-loop may function in conjunction with the GST-like core to facilitate ligand binding.

Here, we use an array of mutants within G-, H-, L-sites and the alpha-loop to reveal that the canonical GST active site houses TA binding activity, while the previously implicated L-site does not. We demonstrate that the alpha-loop plays a direct role in recognizing TA and show that coordination of alpha-loop movement is driven by its contacts with the G-site and that these contacts are required for TA binding. Together, these reveal that coordinated large conformational changes within the canonical active site binding pocket of GDAP1 facilitate TA binding. Even though a direct and specific role for TA in mitochondrial function is unclear, overexpressing wild-type GDAP1, but not TA binding mutants, in human cells reduces mitochondrial oxidative stress and increases mitochondrial membrane potential under resting conditions, suggesting a mitoprotective role for GDAP1 and specifically its TA binding pocket.

## Results

### The GDAP1 Ligandin-site does not play a direct role in interactions with thapsic acid

**Figure 1:**
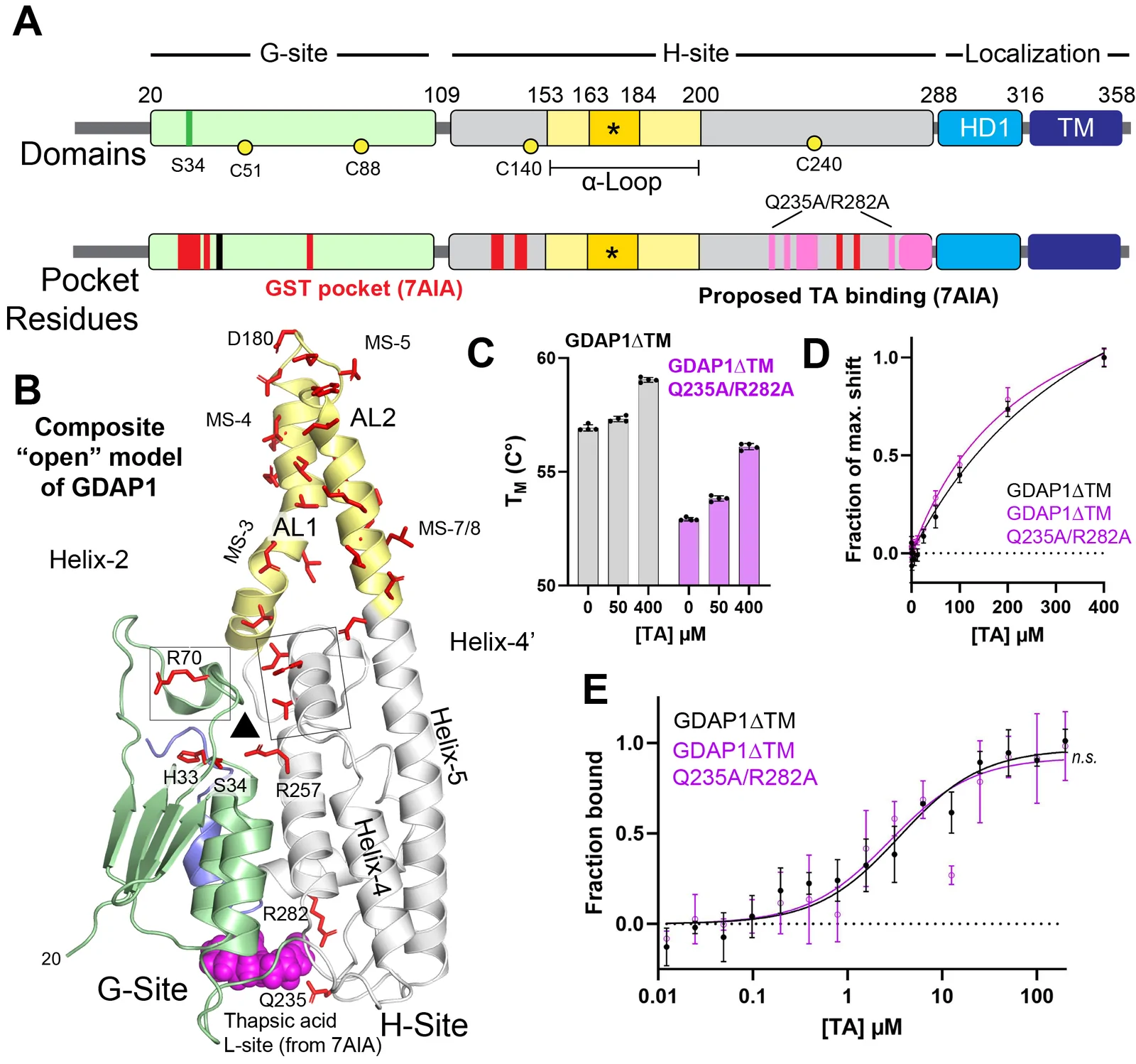
**The GDAP1 L-site does not play a primary role in Thapsic acid binding.***A*, *top*, Domain diagram of GDAP1 based on primary sequence homology with GSTs as well as domain boundaries observed structurally A disordered region within the alpha-loop (*yellow*) is indicated (∗). GDAP1 residues participating in TA interactions (*pink*) or found within the canonical GST binding pocket in 7AIA (*red*) are indicated. *B*, a composite model of GDAP1 structure drawn largely from 7AIA shows the alpha-loop in an extended conformation relative to the primary H-site helices 4 and 5. For clarity, we have modelled the disordered section within the alpha-loop. The remaining positions, including the position of the rest of the alpha-loop and the TA moiety, are unaltered. Residues mutated and tested for TA binding as a part of this study are shown in *red sticks*. Helix-4′ containing the AAA mutation, and G-site helix-2 are boxed to indicate their proximity to the canonical GST binding pocket (*black triangle*). *C*, DSF data demonstrates that TA stabilizes both WT and an L-site mutant (Q235A/R282A). *D*, normalized DSF data shows no significant changes in concentration-dependent stabilization as determined *via* F-test (*p*-value = 0.078). *E*, the same L-site mutant shows no significant impact on TA binding when measured using MST, as determined *via* F-test (*p*-value = 0.43, *n*.*s*.).

Both primary sequence homology (18, 19) and structural analysis (14, 15, 17, 39) have demonstrated that GDAP1 is a novel member of the GST superfamily of proteins. Canonical GSTs contain a GSH-binding G-site and a substrate-binding H-site that fold together to form a large central active site capable of housing both co-substrates simultaneously (Fig. 1, *A* and *B*) (22, 40). GDAP1 also contains an N-terminal extension (amino acids 1-20) of unknown function, and C-terminal Hydrophobic (HD1) and Transmembrane (TM) regions that have been implicated in its membrane localization (16, 41) and an insertion within the H-site, called the alpha-loop, which is unique to GDAP1. The overall domain organization and structure are shown in Figure 1, *A* and *B* (14).

Previous studies of GDAP1’s biochemical activity have been focused on human and mouse proteins which are 94% identical. GDAP1 homologs, however, can be found throughout vertebrates and invertebrate species, albeit with much lower sequence identity, raising the question of whether TA binding was a conserved GDAP1 activity. Therefore, we selected the invertebrate *O*. *sinensis* (East Asian common Octopus) as a model invertebrate, purified its GDAP1 protein recombinantly, and asked whether this octopus GDAP1, which is 30% identical to human GDAP1, retained the ability to interact with TA. This and all other proteins presented in this study were subjected to multiple rounds of purification and exhibited single-state transitions by Differential Scanning Fluorometry (DSF) demonstrating that they were properly folded (Figs. S1 and S2). As shown in Fig. S3, all three GDAP1 homologs were stabilized by the addition of TA in a concentration dependent manner, suggesting that this lipid binding activity may be conserved throughout the animal kingdom.

To test the role of the L-site on TA binding, we made a double mutant Q235A/R282A in human GDAP1 designed to impact two prominent hydrogen-bonding interactions between TA and GDAP1 observed in the previously published structures (15). Both residues are positions of CMT-causing mutants, but neither had been tested biochemically for their role in TA binding. These and subsequent mutants presented here were made within the context of human GDAP1ΔTM (amino acids 20-316) to enable *in vitro* analysis. We first tested the ability of GDAP1ΔTM and the Q235A/R282A L-site mutant to respond to TA using DSF, finding that both proteins were stabilized by the addition of 50 and 400 μm TA (Fig. 1*C*), suggesting an interaction was occurring even with the L-site mutant. To ask whether we could detect a change in binding affinity, we measured stabilization throughout a TA titration (Fig. 1*D*), finding no statistical difference between the titrations (F-test, *p*-value = 0.078). To confirm this unexpected result, we performed TA binding measurements using microscale thermophoresis (MST) (Fig. 1*E*), observing a K_D_ of 3.1 ± 0.8 μm for GDAP1ΔTM, consistent with binding affinities measured by other groups using isothermal titration calorimetry (15). Again, the L-site mutant had no significant impact on binding affinity (K_D_ of 3.7 ± 1.4 μm) in this assay (F-test, *p*-value = 0.43). From these results, we conclude that the L-site does not directly participate in TA binding in solution.

### GDAP1’s G- and H-sites mediate interactions with thapsic acid

**Figure 2:**
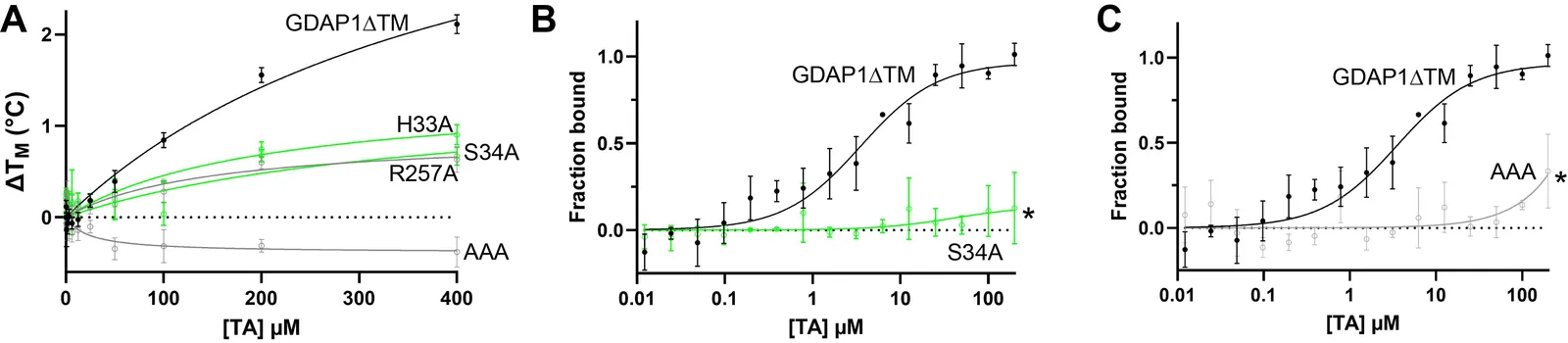
**Mutations in the GDAP1 G-site and H-site impact TA binding.***A*, the stability of the GDAP1-TA complex is reduced when residues in the canonical active site pocket are mutated. T_M_ shifts upon TA titration for indicated mutants as measured *via* DSF. *B* and *C*, Changes in binding isotherms as measured by MST for GDAP1ΔTM and the indicated GDAP1 mutant. Changes in binding affinity were statistically significant as determined *via* F-test (*p*-value <0.0001 indicated by ∗).

Next, we asked whether TA-binding capacity could instead be mediated by GDAP1’s canonical active site pocket, formed by both its G- and H-sites. To accomplish this, we generated three point-mutants in the “floor” of the pocket: H33A, S34A, and R257A (Fig. S4). S34 is at the location of the active site residue in canonical GSTs (23), while the neighboring H33 is important for GST conjugation activity in the liver-specific GST psi (42). R257 is a highly conserved residue in the H-site adjacent to S34, which is critical for conjugation in many GSTs. Given the location of these residues and their roles in substrate interactions within other GST active sites, we asked whether point mutations at these residues would alter TA-mediated stabilization of GDAP1. By DSF, all three mutants showed a dramatic decrease, but not total loss of TA induced protein stability (Fig. 2*A*). Next, we mutated helix-4′, a short helical region which is highly conserved in GDAP1 orthologs and is important for substrate recognition in a variety of GSTs (43, 44). We made a triple mutant (T137A/H138A/I141A, also called AAA here) within helix-4′, which was severely impaired in TA induced stability by DSF (Fig. 2*A*). Next, we used MST to measure TA binding affinity to GDAP1ΔTM, S34A, and the AAA mutants. As shown in Figure 2, *B* and C, these mutants exhibited a significant loss of binding, consistent with a loss of direct interactions between GDAP1 and TA (F-test, *p*-value <0.0001 for both). Together, these observations led us to conclude that regions of the binding pocket that mediate substrate binding in canonical GSTs likewise mediate TA binding interactions in GDAP1.

### Thapsic acid interactions also require the alpha-loop insertion in GDAP1

**Figure 3:**
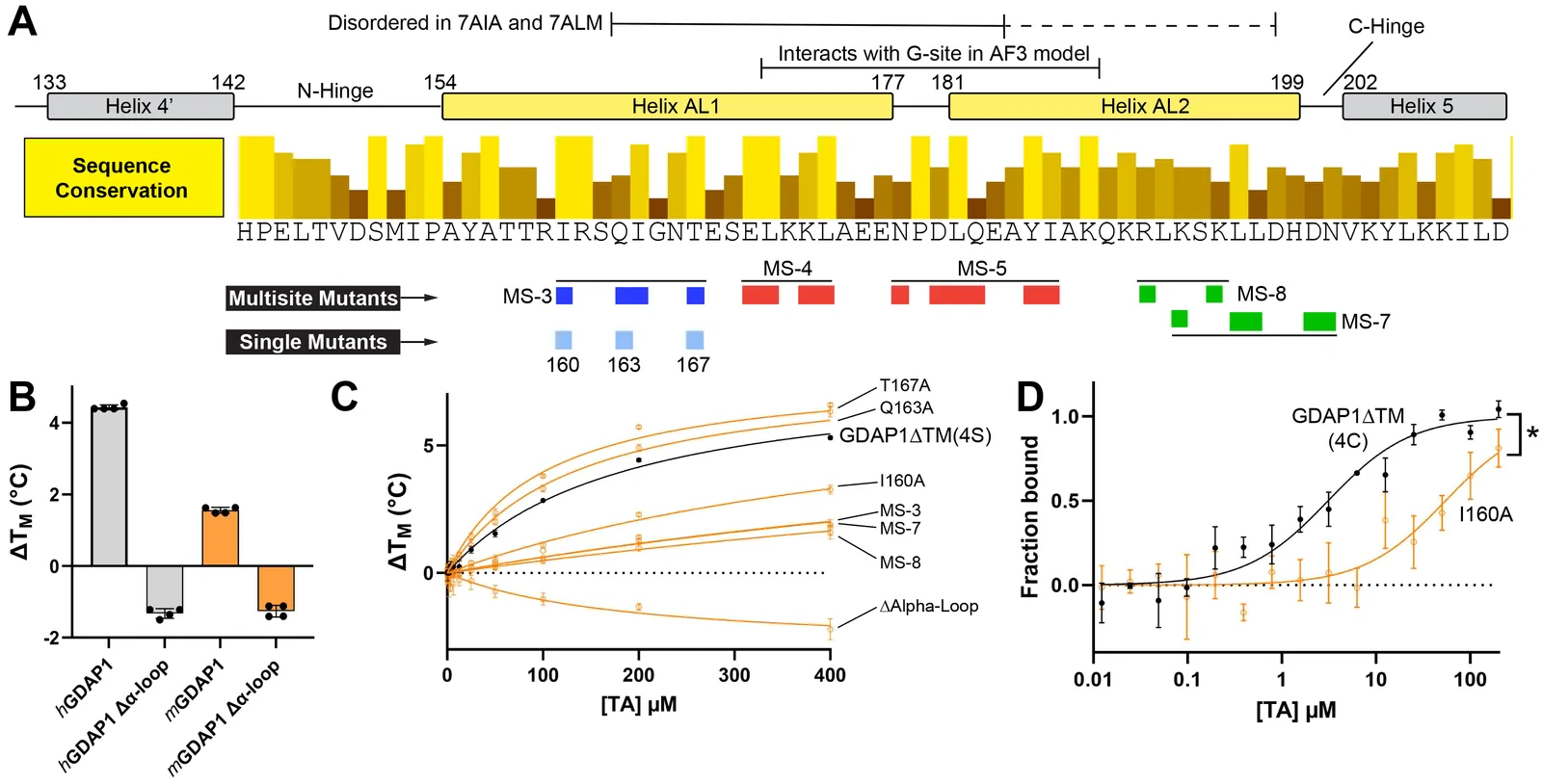
**The GDAP1-alpha-loop is required for TA binding.***A*, diagram of GDAP1’s alpha-loop region, showing the second structural features, sequence conservation within vertebrates, the locations of putative alpha-loop hinge points, and the locations of multi-site and single point mutations tested here. *B*, T_M_ shifts in stability upon addition of 200 μm TA for GDAP1ΔTM and alpha-loop deletions within both human and mouse GDAP1. *C*, stabilization of indicated GDAP1 Alpha-loop mutants and their respective control, GDAP1ΔTM-4S. *D*, binding of TA to GDAP1ΔTM and an I160A mutant measured by MST. I160A yielded significant changes in binding affinity as determined *via* F-test (*p*-value <0.0001, indicated by ∗).

Previous data from our lab had demonstrated that deletion of the alpha-loop in mouse GDAP1 resulted in a loss of binding to the pan-GST inhibitor ethacrynic acid (14). To see if this was also the case for TA, we made an alpha-loop deletion in human GDAP1 replacing amino acids 154 to 200 with a GSGSGS linker (Fig. 3*A*). AlphaFold3 (45) predictions suggest that this linker can replace the alpha-loop without perturbing the rest of the GDAP1 fold. Both mouse and human alpha-loop deletions show a dramatic loss of TA-mediated stabilisation (Fig. 3*B*), suggesting that this region also participates in TA interactions despite not residing in the canonical binding pocket as observed in the structural data (Fig. 1*B*). These findings identify the alpha-loop as an additional motif that is important for interactions with TA.

We next sought to identify residues within the alpha-loop that contribute to TA binding. Guided by both experimental and predicted structures, we designed large multi-site alanine substitutions within the alpha-loop, favoring conserved residues while avoiding changes at positions that might destabilize the protein fold within the alpha-loop itself, notably between putative helices AL1 and AL2, which are predicted to make up a large portion of the alpha-loop (Fig. 3*A*). To also avoid the possibility that disulfide bond formation at residue C88 (14, 15, 17) might influence our ability to detect TA interactions, these multi-site mutations were made within a protein background in which all four cysteines were substituted with serines (GDAP1-4S). We confirmed that the introduction of these serines had no impact on TA binding *via* MST (Fig. S5) and that all proteins were monomeric as observed *via* analytical size exclusion chromatography (Fig. S6). We then tested the impact of alpha-loop mutations on GDAP1 stability throughout TA titration using DSF (Fig. 3*C*). Mutant MS3, which is located deep within the pocket on helix AL1, showed a significant decrease in TA concentration-dependent stability in this assay, indicating that these changes decreased GDAP1’s interaction with TA (Fig. 3*C*). We then followed this up with single-point mutants (I160A, Q163A, or T167A), finding that I160 plays the primary role in TA stability. We then quantified TA binding affinity to the I160A mutant and compared it to GDAP1ΔTM, using the native background containing all four cysteines for both proteins. Here, we observed a significant loss in binding affinity (K_D_ of 54.5 ± 16.1 μm) compared to GDAP1ΔTM (K_D_ of 3.1 ± 0.8 μm) (F-test, *p*-value <0.0001) (Fig. 3*D*). Together, these data identify residue I160 within AL1 as an important mediator of TA binding interactions and help explain why the alpha-loop deletion was deficient in TA binding.

To assess the impact of helix AL2 on TA interactions, we made two multi-site mutants, MS-7 and MS-8 (Fig. 3*A*). Interestingly, the amino acids targeted in these mutants face opposite directions in the existing structural data (Fig. S7), but both had a large impact on concentration-dependent stability *via* DSF, leading us to conclude that helix AL2 is also important for TA interactions. Given the location of the surfaces tested *via* MS-7 and MS-8, it is difficult to assign a mechanistic role for AL2, but it is nonetheless clear that the AL2 helix plays an important role in mediating GDAP1-TA binding. Together with the results testing helix AL1, this demonstrates that the entire alpha-loop participates in ligand binding interactions, and given the proximity of AL1 and AL2, it is reasonable to suggest that they may work together in this capacity as a single functional unit.

### A structural model of the GDAP1-thapsic acid complex

**Figure 4:**
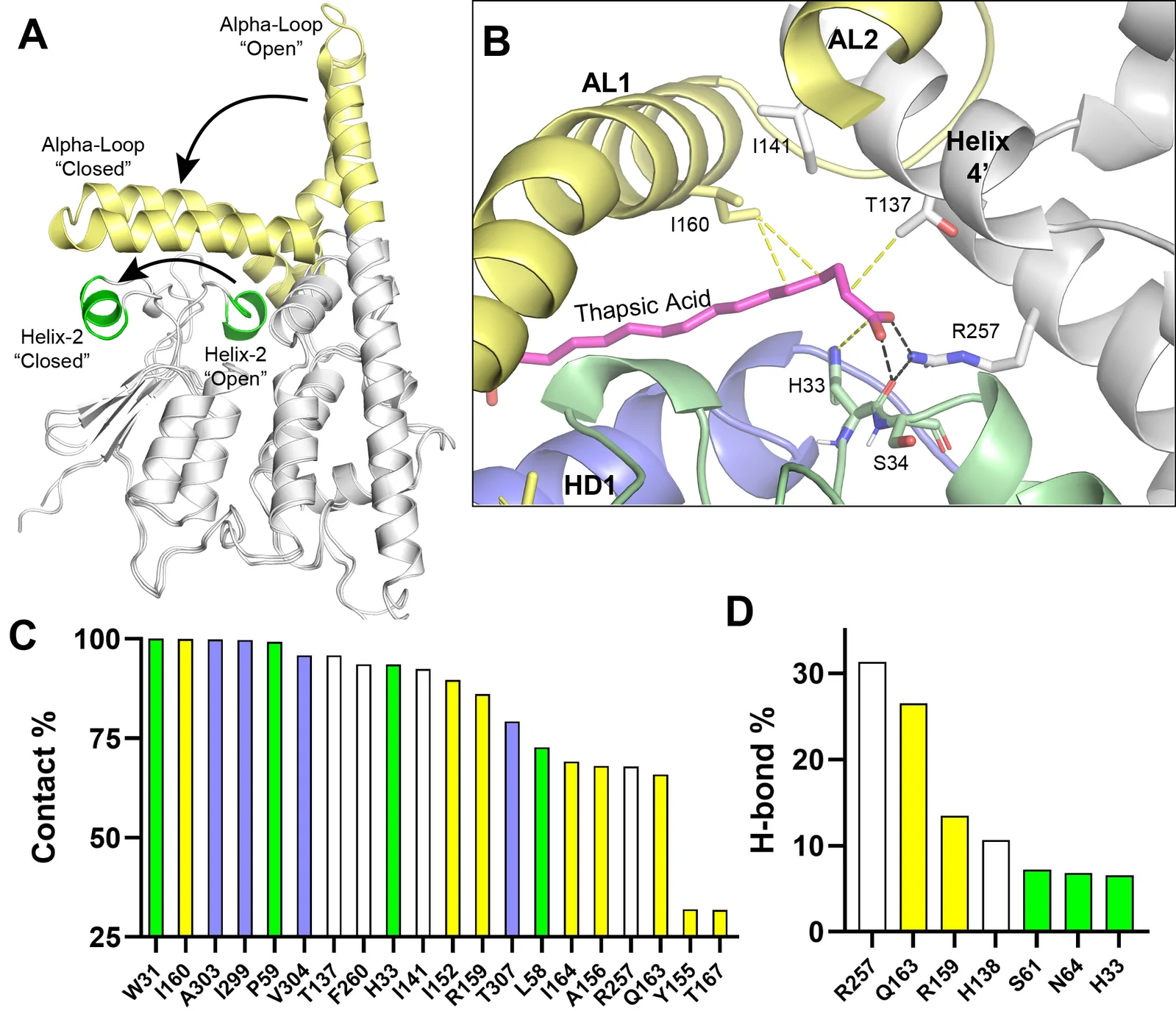
**A model for GDAP1-thapsic acid interactions.***A*, structural overlap of the open (7AIA) and closed (AF3, this study) structures of GDAP1 highlights the conformational changes predicted by the AlphaFold3 model. Shown in *green* is the change in helix-2 positioning where it is shifts out of the active site and creates new contacts with the alpha-loop shown in *yellow*. *B*, the positioning of thapsic acid within the closed state of GDAP1. *Black dotted lines* indicate hydrogen bonding, while dotted *yellow lines* indicate van der Waals contacts. *C*, the percentage of frames in which specific residues are contacting TA over the course of the MD simulation. The *top* 20 GDAP1 residues making contacts with TA are plotted. *D*, hydrogen bond percentages for the *top* 7 residues making H-bonds between their side chains and TA.

Despite being at very distinct sites within the experimental structures, mutations in the G-site, H-site, and alpha-loop demonstrated that each of these domains contributes to TA binding (Figs. 2 and 3). This is incompatible with the existing structural data, so we used AlphaFold3 to predict the binding site and orientation *de novo*. The prediction was performed with five independent seeds, generating a total of 25 models. Model quality was assessed using standard AlphaFold3 confidence metrics, with the top-ranked model having a pTM of 0.80, an ipTM of 0.86, and an overall ranking score of 0.92, indicating high confidence in both the overall fold and the GDAP1-TA interaction. Mean pLDDT was 85 for GDAP1 and 71 for TA, and consistent metrics were obtained across all 25-models, confirming the prediction was reproducible across independent seeds. A structural alignment of the GDAP1-TA complex generated from AlphaFold3 with the previous experimental structure indicates that most of the G- and H-sites are unchanged with r.m.sd values of 0.349 and 0.380 Å, respectively. However, there were dramatic differences in the positioning of the alpha-loop and G-site helix-2 (Fig. 4*A*). The shift of helix-2 away from the TA binding pocket increases the size of the binding pocket from 1102 Å^3^ to 1839 Å^3^ (Fig. S8), and increases solvent accessibility for residues at the floor of the binding pocket (a.a.31-37). The model also suggests a striking pivot in the position of the alpha-loop, which now makes prominent contacts with the newly repositioned G-site helix-2, generating 503 Å^2^ of buried surface area, and forming a lid at the top of the active site binding pocket (Fig. 4*A*). The location of hinge points in this model is consistent with previous predictions (17).

As shown in Figure 4*B*, the prediction for the GDAP1-TA complex derived from AlphaFold3 positioned TA within the canonical active site pocket, broadly consistent with our biochemical results. We next performed 100 ns of MD simulation on the top-ranked model and analyzed to identify residues within GDAP1 that made prominent and stable contacts with TA over the course of the simulation. We began by quantifying the percentage of time within the 100 ns simulation that each amino acid was within 4 Å of TA (Fig. 4*C*). Consistent with our biochemical results presented earlier, we found prominent contacts with residues from every domain in GDAP1 except for the transmembrane helix. There were several notable residues contacting TA from within the alpha-loop, including I160, which also directly impacts TA binding when tested *via* MST. Also prominent are residues within the floor of the active site pocket, including W31, H33, and R257. This is consistent with direct contacts we demonstrate experimentally for S34 and R257 (Fig. 2, *A* and *B*), both located within the floor of the pocket. Additionally, the hydrogen bond formed by R257 is among the most prominent and is consistently found within the timescale of the simulation (Fig. 4*D*). The model predicts contacts between the newly repositioned helix-2 and TA, including residues 59, 60, 61, and 64 and with H-site helix 4′ T137, H138, and I141, which form the AAA mutant, shown to abrogate TA interactions (Fig. 2*C*). Together, these unbiased co-folding predictions are largely consistent with the biochemical data presented and provide a mechanistic lens through which to view the consequence of ligand binding within the GDAP1 active site pocket.

### Disrupting alpha-loop:G-site contacts impair thapsic acid binding

**Figure 5:**
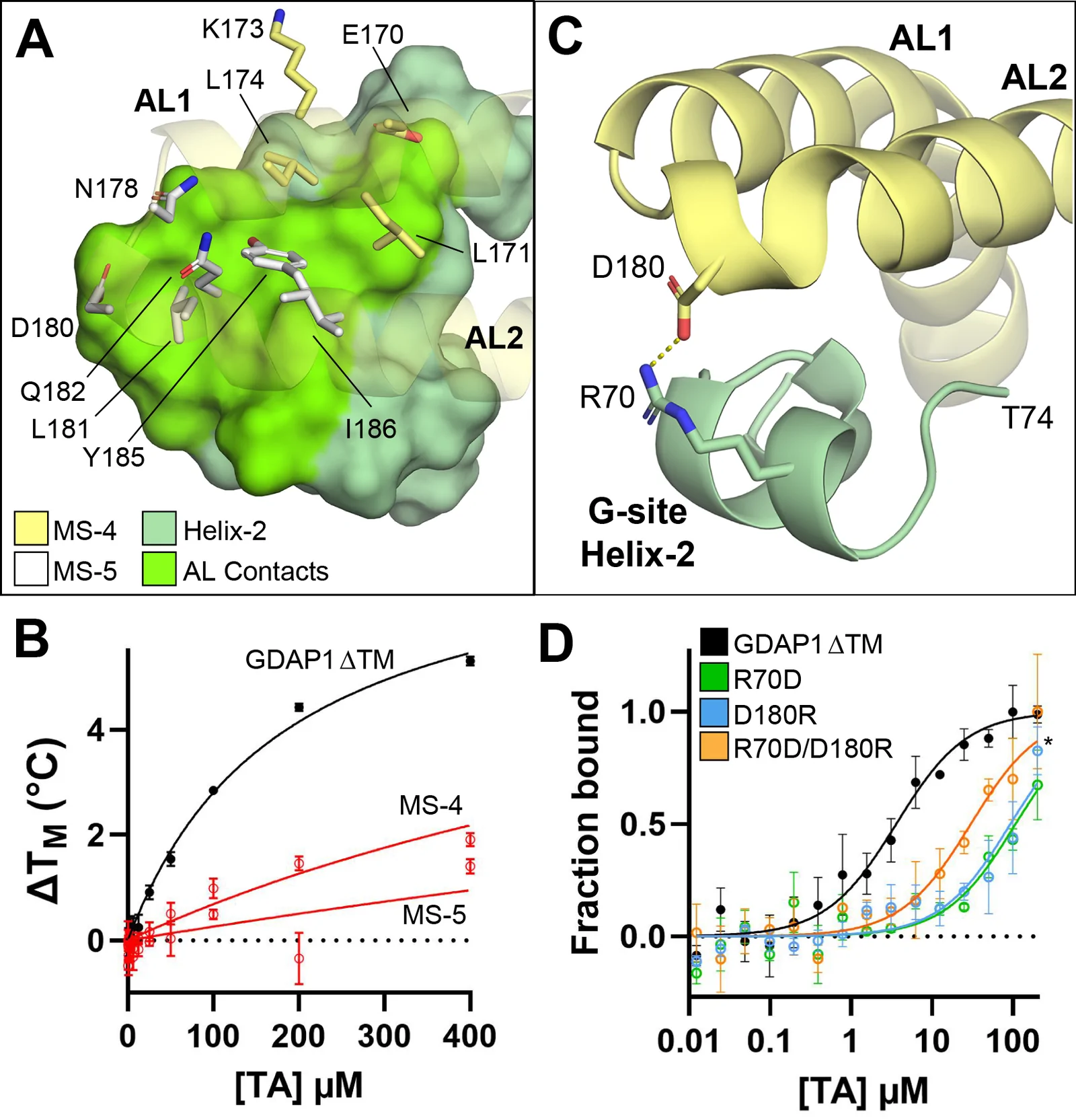
**Disruption of an ionic bond between the alpha-loop and G-site of GDAP1 perturbs interactions with thapsic acid.***A*, the predicted positions of residues mutated in MS-4 (*brown*) and MS-5 (*white*) is shown as sticks. *B*, T_M_ shift of GDAP1-4S and multi-site mutants at the tip of the alpha-loop upon incubation with 200 μm TA. *C*, the positioning of R70 (*green*) and D180 (*yellow*) highlights the salt bridge predicted in the AlphaFold structure. *D*, binding isotherms for GDAP1-4S (*black*), R70D (*green*), D180R (*gold*), and R70D/D180R (*orange*) from MST. The charge-swapped double mutant shows a significant partial restoration of TA binding affinity when compared to either single-point mutant (F-test, *p*-value <0.0001, indicated by ∗).

The biochemical data and AlphaFold predictions suggest that binding within the canonical pocket is facilitated by a transition to a closed-state in which the alpha-loop directly contacts G-site helix-2 and covers the binding pocket. To test this hypothesis, we designed two multi-site mutants (MS-4 and MS-5) at the end of the alpha-loop in helices AL1 and AL2, respectively (Figs. 3*A* and 5*A*). Importantly, these sites are completely exposed within the previous unbound structures and do not line the binding pocket in the AlphaFold prediction (Fig. 5*A*). MS-4 and MS-5 were tested by DSF, resulting in decreased TA-mediated stability for both, but dramatically so for MS-5 in which the stabilizing effect of TA was completely lost (Fig. 5*B*). This suggests that this region in AL2 plays an important role, perhaps by helping to stabilize the interactions between the alpha-loop and helix-2. To test this, we examined the predicted GDAP1-TA complex and found a putative salt bridge between R70 in G-site helix-2 and D180 at the tip of the alpha-loop (Fig. 5*C*). D180 is also one of the residues in the MS-5 mutation, that disrupts TA-mediated stabilization. Using MST, we find a significant decrease in TA binding affinity with the R70D and D180R single mutants (108 ± 29.7 μm and 93.3 ± 24 μm, respectively. F-test, *p*-value <0.0001), demonstrating that these substitutions impair the ability of GDAP1 to bind TA, despite not being located within the canonical binding pocket (Fig. 5, *C* and *D*). Lastly, we asked whether swapping the charges *via* a R70D/D180R double-mutant would be compensatory. Here, we found a significant partial restoration in TA binding affinity (30.2 ± 8.8 μm) (Fig. 5*D*) as compared to either of the single mutants. This is consistent with a model in which we have restored the salt bridge between these two residues, thereby improving the ability of the double-mutant protein’s ability to adopt the closed state relative to either single mutant. It, however, also suggests that additional stabilizing interactions provided by R70 and D180 cannot be restored within the context of the charge-swap double mutant. These data support the identification of residues R70 and D180 as a point of contact between the alpha-loop and G-site and further validate the structural modeling of the GDAP1-TA complex.

### Mutations that alter thapsic acid binding reduce GDAP1-mediated mitoprotection

**Figure 6:**
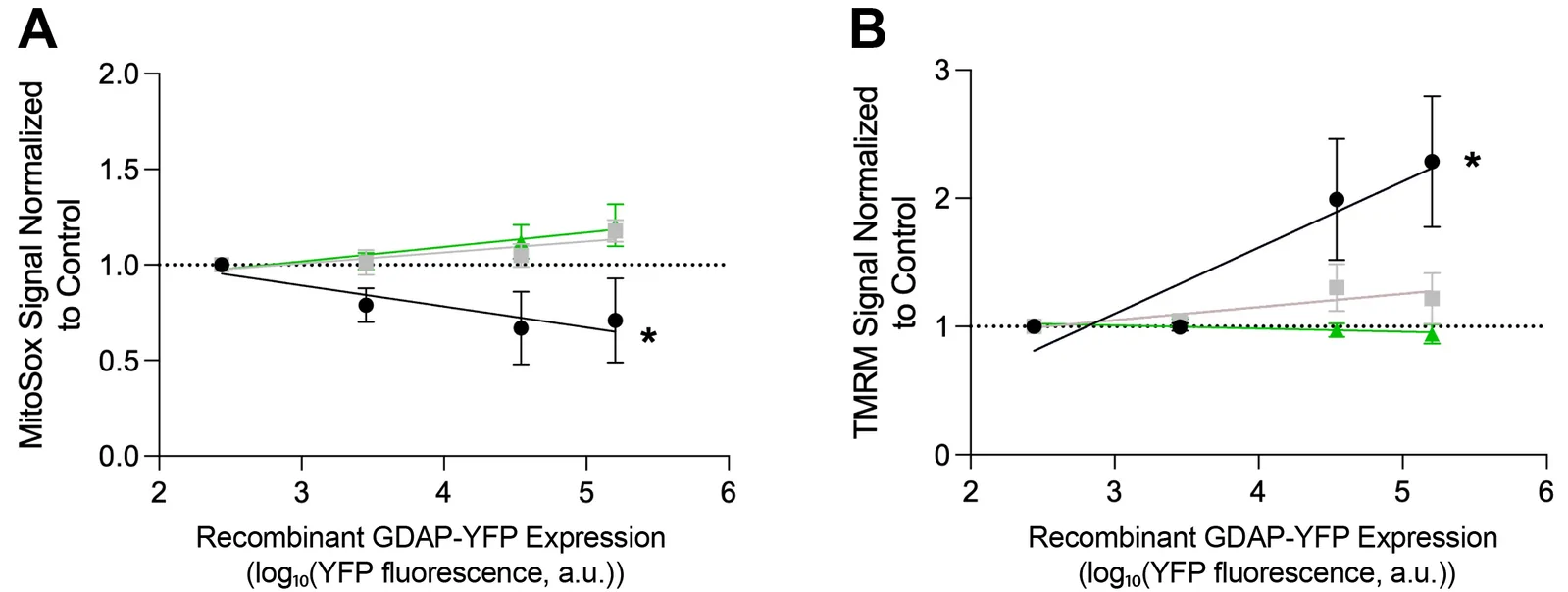
**Wild-type but not TA-binding mutants reduce mitochondrial superoxide and increase mitochondrial membrane potentials.** A summary of flow cytometry experiments (n = 5–10) with MitoSox (*Panel A*) and TMRM (*Panel B*) in HEK293 cells under resting conditions. ∗ Indicates data cannot be fit with the same curve with *p* <0.05 significance. The data were normalized to in-tube GDAP1-negative controls in each experiment.

GDAP1 mutations were previously associated with increased mitochondrial oxidative stress, and here, we used this readout alongside mitochondrial membrane potential measurements to gauge the effects of TA-binding mutations on mitochondrial health. Figure 6 shows the results of flow cytometry experiments with mitochondrial membrane potential dye TMRM and mitochondrially targeted superoxide marker MitoSOX (46) in HEK293 cells transiently transfected with YFP-tagged human wild-type or YFP-tagged versions of GDAP1, AAA, and H33A mutants. Based on the average YFP fluorescence, the cells were binned into control (no recombinant GDAP1 expression, leftmost points in Fig. 6, *A* and *B*), and low, medium, and high recombinant GDAP1 expression. The amount of TMRM or MitoSOX fluorescence was recorded under resting conditions. The no-YFP cells served as in-tube controls against which TMRM and MitoSOX fluorescence across all expression groups were normalized, revealing GDAP1 effects. The downward deflection of the MitoSOX signals indicates lower mitochondrial peroxide levels compared to no-YFP controls, and the upward deflection of the TMRM signal indicates increased mitochondrial membrane potential relative to the in-tube control. Figure 6 shows that cells overexpressing WT GDAP1 have lower mitochondrial superoxide levels and higher membrane potential than control cells or cells overexpressing TA binding mutants, supporting a mitoprotective function of GDAP1 which is lost when the residues critical to substrate binding in the classical GST active site are altered and higher membrane potential than control cells or cells overexpressing TA-binding mutants, supporting a mitoprotective function of GDAP1 which is lost when the residues critical to substrate binding in the classical GST active site are altered.

## Discussion

Multiple crystallographic analyses from our group and others have demonstrated that GDAP1 can adopt an “open” structure in which the alpha-loop extends H-site helices 4 and 5, leaving the top of the GST-like canonical binding pocket open (Fig. 4*A*). The alpha-loop has been shown to be required for binding of the pan-GST inhibitor ethacrynic acid (14) and TA (this study), a finding that challenges the previous structural data describing GDAP1-TA interactions. This crystal form was promoted through extensive crystal contacts mediated by helices AL1 and AL2 of the alpha-loop with a combined interface area of over 1100 Å^2^. We noted that both the apo- and TA-bound structures were crystallized in similar conditions, which may have prevented the alpha-loop from adopting the closed state within the crystal lattice in the presence of TA. Subsequent structural data (without TA) and structural predictions suggested that positioning of the alpha-loop and G-site helix-2 were not uniform (14, 15, 16, 17). Together, these observations motivated our hypothesis that these regions mediate GDAP1-TA interactions. Analysis of an array of mutants within G-, H-, and alpha-loop domains demonstrates that the bound state of GDAP1 must adopt a conformation that is significantly different from those previously observed crystallographically and is consistent with the closed state predicted by AlphaFold. Our biochemical analysis here is the first to interrogate the closed state of GDAP1 and establish its connection with ligand binding. GST-family members are known for recognizing a variety of related substrates within their active sites, and our studies here add to this collection while also establishing a new mechanistic paradigm for their interactions within GDAP1. Future studies will be required to identify other lipid-binding partners for GDAP1 and to establish a molecular mechanism for selectivity within the canonical binding pocket.

The presence of CMT mutations within the L-site pocket does suggest that it is a functionally important location on the protein. With the data presented here, the functional role of this site is currently unclear, but its proximity to the membrane interface would make it available to interact with other lipids or proteins within the outer mitochondrial membrane. It is also possible that the L-site is serving as an allosteric or secondary binding site. Additional data establishing the biological role of the L-site will be critical in establishing the impact of this site.

Decades of documenting missense mutations in GDAP1 have identified >70 missense mutations that cause CMT (10, 47, 48, 49). These CMT-causing mutations are located within all of GDAP1’s important protein domains: G-site, H-site, alpha-loop, HD1, and transmembrane anchor. Mutants in the transmembrane domain disrupt mitochondrial localization (41), while many other CMT mutants are presumed to impact GDAP1 stability, folding, or protein turnover (39). Additionally, there is a previously identified cluster of surface exposed mutants within the H-site known as the CMT hotspot (14, 17). A function for this region has not been firmly established, although it functions as the primary dimerization interface for canonical GSTs (14, 28) suggesting it may function as a protein interaction surface for an as yet unidentified binding partner. Interestingly, R70 and D180 are not currently annotated as CMT causing mutants, however, there are CMT mutants in the contact patch between the G-site and the alpha-loop, including N178 which contacts G-site residue P66. Our data suggests that dysfunction caused by these mutants may be the result of a diminished capacity to achieve the closed state. To date there is not a proven mechanism unifying the role of mutants in the binding pocket with those of CMT hotspot; however, structural studies have detected a network of interconnected interaction between disease-causing residues (17). Thus, it is plausible that conformational changes in GDAP1 resulting from ligand binding may impact the adjacent CMT hotspot. Such a mechanism would explain why GDAP1 appears to have limited or no enzymatic activity, supporting the hypothesis that it functions as a molecular sensor or signaling protein.

In this study, we demonstrate that disruption of a salt bridge between R70 and D180 dramatically impacts ligand binding, likely by interfering with the ability of GDAP1 to adopt the closed ligand-bound state. These experiments demonstrate for the first time a direct contact between Helix-2 and the alpha-loop and suggest that binding within the canonical GST pocket may result from coordinated movements between these two mobile elements. Interestingly, we have identified other GST-like proteins, including the plant-specific TCHQD class, DCHQ-D (also known as LinD) from the soil bacteria *Sphingobium indicum*, and vertebrate Microsomal prostaglandin E synthase type-2, which share structural homology with GDAP1’s AlphaFold prediction. It suggests that other distantly related proteins within the family also contain alpha-loops, and hints that these may share a common mechanism for binding which is distinct from canonical GSTs.

The logic of GDAP1 regulating stress is premised on GDAP1’s homology to GSTs, many of whom are regulated by oxidative stress and are involved in oxidative stress responses (26, 50) as well as evidence connecting GDAP1 expression to oxidative stress (12, 51, 52). While the individual pieces of evidence are compelling, they should be taken with caution as an argument for a specific function, given the broad range of cellular processes associated with oxidative stress (12, 26, 53). For example, dysregulation of mitochondrial fusion/fission elements DRP1 and MFN2 is linked to oxidative stress, although neither protein has the capacity to directly regulate reactive oxygen species (54, 55). The evidence presented here, combined with reports from multiple groups that GDAP1 does not bind glutathione, suggests that GDAP1 does not directly regulate mitochondrial oxidative stress using any canonical GST-like activity, but likely does so indirectly through shape regulation or by modulating mitophagy, as was recently proposed (56).

Taken together, the evidence suggests that GDAP1 plays a protective role against cell stress, with the mechanism remaining to be described. Our data clearly show that GDAP1 possesses a functional binding pocket, and that perturbation of this binding site impacts levels of oxidative stress. These data support either direct or indirect roles for the GDAP1 active site in regulating ROS levels and mitochondrial membrane potential. Further work is needed to determine a molecular mechanism for GDAP1 that unifies these biological functions. However, this is the first connection between biochemical and cellular functions for GDAP1, advancing our understanding of CMT pathogenesis of GDAP1 mutations in or around the active site. Further characterization of the mechanism connecting GDAP1 to its role in oxidative stress and mitochondrial morphology is required, but this is an important step forward in developing a molecular understanding of CMT pathogenesis.

## Experimental procedures

### Cloning, expression, and purification

The codon-optimized coding sequence corresponding human GDAP1 (a.a. 21-315) (UniprotKB: Q8TB36) were synthesized and cloned into the pet28 vector by TWIST Biosciences. Protein expression and purification was performed as described previously (14). Protein purity after purification was monitored using SDS-PAGE with Coomassie staining and the final products were >99% pure (Fig. S1). Gels were imaged using trans-illumination on an Amersham Imager 600. Adjustments to brightness were made in Image Lab (BioRad) and were made on the entire image prior to splicing. Size exclusion chromatography was performed on a Superdex 200 HR 10/30 column at a concentration of 1.5 mg/ml in 200 mm NaCl, 20 mm Tris pH 8, 2% glycerol, 1 mm TCEP demonstrated that our protein was monomeric, both for GDAP1ΔTM constructs containing the native four cysteine residues (4C) as well as when those residues are mutated to serines (4S) (Fig. S6). Mouse (a.a. 1-322) and Octopus (a.a. 1-310) GDAP1 were expressed and purified as their human counterpart.

### Differential scanning fluorimetry

DSF assays were conducted using a Thermo-Fisher QuantStudio 3 Real-Time PCR machine. Using the 96-well plate format, 20 μl reactions were performed in at least triplicate at a final protein concentration of 0.4 mg/ml. Reactions were carried out at a final concentration of 100 mm HEPES pH 7.5, 50 mm NaCl. GloMelt was used to monitor temperature-dependent unfolding at a final concentration of 0.5x using an excitation wavelength of 468 nm. Fluorescent emission at 507 nm was measured as the samples were exposed to a temperature gradient of 30 to 95 °C and the melting temperature (T_M_) for each sample was defined by the peak of the first derivative curve. For assays with hexadecanedioic acid (thapsic acid; Sigma-Aldrich) and/or GSH (ThermoFisher), stocks were generated in DMSO (TA) or water (GSH). Ligands were added to the reaction mixture at a final volume of 5% (v/v%). Control reactions contained vehicle at a final volume of 5% (v/v%). For reactions containing both TA and GSH (or reductant; both suspended in water), each was added at a final volume of 5% (v/v%).

### Cell culture

HEK293 cells for flow cytometry were grown at 37 °C and 5% CO_2_ in Dulbecco Modified Eagle’s Medium DMEM (ThermoFisher) supplemented with 10% fetal bovine serum (ThermoFisher). The cells were grown in culture-treated Petri dishes or glass coverslips for imaging. Transfections were performed using Lipofectamine, and cells were harvested for experiments 24 to 48 h post-transfection.

### Cell biological assays

For flow cytometry, cell monolayers were enzymatically digested using Tryple (ThermoFisher) and resuspended in HEPES-based medium containing, in mm: 140 NaCl, 5 KCl, 1 CaCl_2_, 1 MgCl_2_, 10 HEPES (pH 7.4), and 1 g/l glucose. The cells were transfected with YFP-tagged WT or mutant GDAP1 or with an empty vector. The cells were loaded with 0.1 μm MitoSox or Mitotracker (ThermoFisher) for 15 min at 37 °C. 10 thousand cells were analyzed per sample. To identify cells expressing recombinant YFP-tagged GDAP1, cells were analyzed using fluorescence signals recorded with the BL1 filter setting of the Attune N ×T (ThermoFisher) and binned into groups based on median YFP fluorescence (horizontal axes in Fig. 6 show log_10_ values of BL1 signal). Floreada (https://floreada.io) was used to analyze the samples. Samples that were not transfected or loaded with the dye were used as autofluorescence controls. Cells whose fluorescence was indistinguishable from that of control samples were considered not transfected. The binning schema and flow cytometry settings were maintained across experiments. To quantify MitoSox or Mitotracker fluorescence, we calculated the median YL1 signal within each GDAP1 expression bin. The resulting values within each bin were then normalized to the value recorded in the bin with the lowest YFP signal, presumed to be from cells not expressing exogenous GDAP1.

### Microscale Thermophoresis

Purified GDAP1ΔTM or indicated mutants were labeled with NHS-fluorescein according to manufacturer’s instructions (Thermo Scientific). After labeling for one hour at room temperature, the remaining free label was removed *via* sequential dialysis against 3 × 2 L of [20 mm HEPES pH 8, 200 mm NaCl, 5% glycerol, 1 mm TCEP]. Sample reactions were performed in 20 mm HEPES pH 8, 50 mm NaCl, 5% glycerol, 0.25 mm TCEP, and 0.1% CHAPS. 2-fold serial dilutions of thapsic acid in DMSO were prepared in Eppendorf tubes and added to the final reaction at 5% v/v. Labeled GDAP1 concentration was constant for all constructs tested at 30 nm. Samples were loaded into capillaries and MST was performed with a NanoTemper monolith NT.115, using 20% LED power and medium MST power. Data was analyzed using the MO Affinity Analysis software (NanoTemper), using the “K_D_” model and plotted in Prism, consistent with previous analysis of protein-lipid binding (57, 58). Data points which were flagged as aggregation of the MO software were removed from the analysis.

### AlphaFold3 structure prediction

The structure of the full-length GDAP1 monomer (UniProt Q8TB36, 358 residues) in complex with thapsic acid (SMILES: C(CCCCCCCC(=O)O)CCCCCCC(=O)O) was predicted using AlphaFold 3 (45). The ligand binding pose was predicted without any spatial or pocket constraints, allowing AlphaFold3 to determine the binding site and orientation *de novo*. Five random seeds were used, each generating five samples, for a total of 25 predicted models. AlphaFold3 was run *via* Singularity (59) using the official container image with pre-downloaded model weights and genetic databases. Predictions were performed on an NVIDIA L40 GPU. Model quality was assessed using the AlphaFold3 ranking score; the top-ranked model (ranking score 0.92) was selected for downstream molecular dynamics simulation.

### Molecular dynamics simulation with AMBE

The top-ranked AlphaFold3 predicted complex was used as the starting structure for molecular dynamics (MD) simulation in AMBER 24 (60). The protein was processed with pdb4amber and modeled with the ff15ipq force field (61). The ligand was parameterized with the General AMBER Force Field (GAFF) using antechamber for atom typing and AM1-BCC charge assignment. The complex was solvated in a truncated octahedral TIP3P water box with a 12 Å buffer and neutralized with Na^+^ and Cl^-^ counterions.

The system was equilibrated through two-stage minimization, NVT heating to 300 K with positional restraints, and NPT density equilibration, followed by 100 ns of NPT production MD at 300 K and 1 atm (Langevin thermostat, γ = 1.0 ps^−1^; Berendsen barostat, τ_p_ = 2.0 ps) using pmemd.cuda. Coordinates were saved every 10 ps, yielding 10,000 frames for analysis. Solvent and ions were stripped prior to analysis.

Ligand-protein contacts were computed per frame using a 4.0 Å heavy-atom distance cutoff. Hydrogen bonds were identified using MDAnalysis (62) with a donor–acceptor distance cutoff of 3.5 Å and a D–H···A angle cutoff of 120°.

## Statistical analysis

All bar graphs and plots presented were the means and standard deviations of at least three technical replicates. When appropriate, all data sets were tested for statistical outliers using the ROUT method within Prism10. Nonlinear regression curves were fit in Prism 10 software using the one-site specific binding model (GraphPad). For normalized DSF binding curves and MST experiments, nonlinear regression curves were compared using the F-test with a *p*-value cutoff of 0.05 for significance.

## Data availability

The data that support the findings of this study were obtained by analyses described in the methods. All data and plasmids used in this manuscript are available upon request to the corresponding author.

## Supporting information

This article contains supporting information.

## Conflict of interest

The authors declare that they have no conflicts of interest with the contents of this article.
