# Supplementary material for: Conformational plasticity in GDAP1’s GST-like domain enables thapsic acid recognition
Source: J Biol Chem. 2026 Jul 28;302(9):113378. doi: 10.1016/j.jbc.2026.113378 (PMC13524606; doi:10.1016/j.jbc.2026.113378)
Supplement: Supplementary file 1 [file mmc1.docx]

**Conformational plasticity in GDAP1’s GST-like domain enables thapsic acid recognition**

Nicholas T. Arredondo, Michael A. DaSilva, Maya R. Brown, Marios A. Gavrielatos, Sarah M. Helsel, Matthew R. Googins, Zoe Freyberg, David R. Koes, Kirill I. Kiselyov, and Andrew P. VanDemark

Department of Biological Sciences, University of Pittsburgh, Pittsburgh PA 15260, USA

# **Supporting Information**

 **Figure S1**: **Purified mutant GDAP1 proteins used for Biochemical analyses.** Purification of recombinant WT or mutant GDAP1 proteins was performed using nickel affinity chromatography. The resulting proteins used in this study are shown using SDS-PAGE on either 12% or 15% polyacrylamide gels. Arrowheads on the left denote MW markers from each gel, and arrowhead on the right denotes purified GDAP1 constructs at the correct relative position to protein standards run on the same gel (not shown).

**Figure S2:** **Single-state unfolding as measured by DSF for recombinant GDAP1 and GDAP1 mutants used in this study.** Mutants are separated by their respective background (containing the native 4 cysteines [4C], or a background in which each of these have been mutated to serine [4S]) to mitigate any impact from potential GDAP1 disulfide formation. Non-human GDAP1 proteins are shown in red. The plot is the average of a minimum of 3 technical replicates with error bars representing the standard deviation of those measurements.

**Figure S3.** **Vertebrate and invertebrate GDAP1 retain TA binding.** **A)** Single state unfolding curves for *h*GDAP1∆TM, measured via DSF with (red) and without (black) 400 μM thapsic acid. The location of the T_M_ is taken as the peak of the derivative curve as shown. **B)** Concentration-dependent shifts in GDAP1 stabilization upon TA titration. *h*GDAP1∆TM exhibited single-state unfolding transitions and were responsive to the addition of TA in a concentration dependent manner. **C)** Concentration dependent TA induced stability for *mouse* GDAP1 (a.a. 1-322) measured using DSF. **D)** The AlphaFold3 predicted structure of *m*GDAP1. N-terminal extension and TM domains are omitted for visual clarity. **E)** Concentration dependent TA induced stability for *octopus* GDAP1 (a.a. 1-310). **F)** The AlphaFold3 predicted structure of *o*GDAP1. Domains are colored based on the domain boundaries in human GDAP1. N-terminal extension and TM domains are omitted for clarity.

**Figure S4: H33, S34, and R257 compose the bottom of GDAP1’s conserved GST binding pocket.** The region surrounding the canonical binding pocket from PDBid 7ALM. Domains are colored as in Figure 1A with residues H33, S34, and R257 which are located on the “floor” of the binding pocket are shown in red sticks. A dotted line indicated the disordered portions from G-site Helix-2, while the disordered portions of alpha-loop helices AL1-2 are omitted for clarity. The location of the GST-like canonical binding site is boxed for reference.

**Figure S5: GDAP1-4S retains WT binding affinity.** To avoid any effects from disulfide bonds formation within GDAP1, we expressed and purified GDAP1ΔTM-4S, in which the four native cysteines were substituted with serines. The protein was purified and behaved the same as the cysteine containing native version. Here, TA binding affinity was measured using MST in a TA titration. The K_D_ was determined to be 3.7 ± 0.8 μM for this interaction, demonstrating that the cysteines do not participate directly or indirectly in interactions with TA.

**Figure S6: Analytical size exclusion traces of GDAP1 proteins used in this study indicate all protein samples are monomeric.** GDAP1 protein samples (Black: GDAP1 4C, 1 mM TCEP, Red: GDAP1 4S, 1 mM TCEP, Blue: GDAP1 4S, no TCEP). were loaded onto a Superdex 200 HR 10/30 at a concentration of 1.5 mg/mL in 200 mM NaCl, 20 mM Tris pH 8, 2% glycerol, 1mM TCEP. GDAP1 has been described as a dimer mediated in part by a disulfide bond formed by residue C88 from each subunit within the dimer. Here, we find no meaningful differences between the elution profiles of GDAP1 with 1mM of the reductant TCEP, required for solubility, and an alternative variant of GDAP1 in which all of the cysteines have been substituted by serine (GDAP1-4S).

**Figure S7: The environment surrounding Multi-Site mutants MS-7 and MS-8.** Cartoon from PDBid 7ALM showing the region of multi-site mutants MS-7 (yellow sticks) and MS-8 (green sticks) within the GDAP1 open state. MS-8 residues R191 and K195 are pointed towards the right and away from the viewer, while MS-7 residues (K193, L196, L197, D200, and N201) (Yellow) are facing towards the viewer. Helix-AL2 residues 186-189 are hidden for clarity, while disordered residues in helices AL1-2 are shown as a dotted line. Mutants MS-7 and MS-8 are both located outside of the canonical pocket within this structure.

**Figure S8: Fpocket analysis shows the GST-like canonical binding pocket of GDAP1 has a greater volume in the “closed”, AlphaFold predicted structure.** **Top)** Fpocket analysis of the conserved GST-like binding pocket of GDAP1 for both open (7ALM and 7AIA) and closed states (AlphaFold3). We considered the open state both with and without TA bound as well as a composite model in which the conformation of disordered residues 166-198 were added using AlphaFold3 but refined using COOT. In this composite model, the ordered portion of GDAP1 protein was unaltered from 7AIA. **Bottom)** The volume and location of pockets from the open and closed states visualized as red spheres generated by Fpocket.
